# An improved environmental DNA assay for bull trout (*Salvelinus confluentus*) based on the ribosomal internal transcribed spacer I

**DOI:** 10.1371/journal.pone.0206851

**Published:** 2018-11-06

**Authors:** Joseph C. Dysthe, Thomas W. Franklin, Kevin S. McKelvey, Michael K. Young, Michael K. Schwartz

**Affiliations:** U.S. Forest Service, National Genomics Center for Wildlife and Fish Conservation, Rocky Mountain Research Station, Missoula, Montana, United States of America; University of Hyogo, JAPAN

## Abstract

The majority of environmental DNA (eDNA) assays for vertebrate species are based on commonly analyzed regions of the mitochondrial genome. However, the high degree of mitochondrial similarity between two species of charr (*Salvelinus spp*.), southern Dolly Varden (*S*. *malma lordii*) and bull trout (*Salvelinus confluentus*), precludes the development of a mitochondrial eDNA assay to distinguish them. Presented here is an eDNA assay to detect bull trout based on the first ribosomal internal transcribed spacer (ITSI), a nuclear marker. This assay successfully detects bull trout and avoids detecting Dolly Varden as well as brook trout (*S*. *fontinalis*), Arctic char (*S*. *alpinus*), and lake trout (*S*. *namaycush*). In addition, this assay was compared with an extensively used mitochondrial bull trout assay and it was found that the ITSI-based assay produced higher detectability. Our results suggest this assay should out-perform the published mtDNA assay across the range of bull trout, while the added specificity allows reliable bull trout detection in areas where bull trout co-occur with other charr such as Dolly Varden. While clearly a superior assay in this instance, basing assays on ITSI is not without problems. For vertebrates, there are fewer ITSI sequences available than commonly sequenced regions of the mitochondrial genome. Thus, the initial *in silico* screening of candidate assays must be preceded by much more extensive sampling and sequencing of sympatric or closely related taxa. Further, all copies of the internal transcribed spacers within an individual may not be identical, which can lead to complications. Lastly, the copy number for ITSI varies widely across taxa; the greater detectability associated with this assay cannot be assumed for other species.

## Introduction

Bull trout (*Savelinus confluentus*) is a native species of conservation concern that declined dramatically during the 20^th^ century and is now accorded federal protection in the U.S. and Canada [1, 2]. Bull trout are widely distributed but seldom abundant in streams, and traditional protocols [3] are time consuming and expensive to implement for broad-scale monitoring. To provide a more cost effective approach to locate bull trout, Wilcox et al. [4] developed an environmental DNA (eDNA) assay for bull trout in the mitochondrial cytochrome b gene (*cyt b*). This assay proved successful for local [5] and range-wide [6] assessments of bull trout habitat occupancy, but it had several limitations. First, it did not clearly distinguish bull trout DNA from that of lake trout (*S*. *namaycush*) without the use of a blocking primer [7], which increased analytical costs for samples from areas that might have lake trout. Second, bull trout and southern Dolly Varden (*S*. *malma lordii*) exhibit little mitochondrial divergence, presumably due to Pleistocene hybridization during co-occupation of a southerly glacial refugium [8]. Thus, positive detections with the *cyt b* assay could indicate the presence of bull trout or southern Dolly Varden where their ranges overlap in western Washington. Further, the *cyt b* assay may result in a false negative if lake trout DNA is present in high concentration in the sample relative to bull trout DNA, and a lake trout blocking primer is not used. To address these deficiencies, an alternative assay based on a genomic region exhibiting substantial divergence between the aforementioned lineages as well as most other species, but containing multiple copies per cell to enhance eDNA-based species detection was necessary.

Mitochondrial genes are popular targets for eDNA assays because of both high copy numbers and an abundance of available reference sequences at commonly sequenced regions such as *cyt b*. Most nuclear genes are limited to two copies per cell, causing them to be too scarce to provide the high probability of detection associated with most eDNA sampling [9], and are generally avoided as the basis for sensitive eDNA assays. The first ribosomal internal transcribed spacer (ITSI), however, may often have hundreds to thousands of copies per cell [10]. This region is often used for phylogenetic studies because it tends to show relatively large interspecific and sometimes intraspecific differences; such differences are evident between coastal bull trout and southern Dolly Varden [11]. Moreover, this region has been the target of effective eDNA assays for other fish species, specifically common carp *Cyprinus carpio* and Macquarie perch (*Macquaria australasica*) [12–13]. Minamoto et al. [12] also suggested that the higher copy numbers might result in assays with higher target species detection rates than those targeting mitochondrial sequences. Here, a marker for bull trout was developed in ITSI that will identify bull trout in the presence of lake trout, Dolly Varden, brook trout (*S*. *fontinalis*), and Arctic char (*S*. *alpinus*). This assay was then tested against the *cyt b* assay developed by Wilcox et al. [4] to determine the relative sensitivities and target species detection rates of these two assays.

## Methods

We designed a quantitative PCR (qPCR) assay to detect a short fragment of the bull trout ITSI gene using a TaqMan^TM^ minor-groove-binding probe (Life Technologies Corporation, Grand Island, NY, USA). These assays have proved extremely sensitive, generally amplifying >95% of the time when more than 10 copies are present [4, 14] and can be made highly specific [4]. These assays here have the additional benefits of allowing the relative detectability of the assays to be formally compared. To design an eDNA ITSI assay for bull trout, genetic sequence data of this region was compiled for bull trout and 14 other salmonid species (Table 1). Given the limited public sequence data available for this region, additional sequences were generated from DNA extracted from fin clips of Arctic char (*n* = 1), brook trout (*n* = 10), bull trout (*n* = 8), Dolly Varden (*n* = 8), and lake trout (*n* = 3; S1 Table). All tissue and DNA samples used in this study were obtained from archived samples collected under appropriate sampling permits for previous studies. As such, approval by an animal ethics committee was not required. The bull trout, Dolly Varden, and lake trout tissues from Washington were provided by the U.S. Fish and Wildlife Service’s Abernathy Fish Technology Center (Longview, WA) and were stored in ethanol. During DNA extraction, the surface of the tissue was first cleansed of foreign DNA by carefully blotting it with 10% bleach using a Kimwipe. All surfaces of the tissue were then immediately and thoroughly rinsed with distilled water, dried on a clean Kimwipe, and the tissue was extracted using the DNeasy Blood & Tissue Kit (Qiagen, Inc. Valencia, CA, USA) following the manufacturer’s protocol. PCR products for sequencing were amplified using the ITSI primers (forward: 5’ AAAAAGCTTTTGTACACACCGCCCGTCGC 3’; reverse: 5’ AGCTTGCTGCGTTTCTTCATCGA 3’) and cycling conditions (30 cycles of [94°C for 1.5 min, 55°C for 2 min, and 72°C for 3 min] followed by a final extension at 72°C for 7 min) described in Pleyte et al. [15], and were cleaned using ExoSAP-IT™ PCR Product Cleanup Reagent (Life Technologies, Grand Island, NY, USA). Sequences were generated on an ABI 3730XL sequencing machine at Eurofins Genomics (Louisville, KY), were processed in Sequencher v 5.4.6 (Gene Codes Corporation, Ann Arbor, MI, USA), and then were trimmed to the approximately 600-nucleotide ITSI region (S1 Table)

**Table 1:** Species, sample size (*n*), and GenBank accession number for DNA sequences used for *in silico* development of the bull trout ITSI eDNA assay. Also included is the minimum number of mismatches with the assay and the sequence data.

				Mismatches		
Species name	Common name	*n*	GenBank accession	Forward primer	Reverse primer	Probe
*Salvelinus confluentus*	Bull trout	2	AY125170.1; M94902.1	0	0	0
*Salvelinus alpinus*	Arctic char	3	AF059898.1; FJ945338.1; M94901.1	3	6	1
*Salvelinus alpinus alpinus*		4	AF059893.1; AF059894.1-AF059896.1	3	6	1
*Salvelinus alpinus erythrinus*		1	AF059897.1	3	6	1
*Salvelinus fontinalis*	Brook trout	1	M94903.1	3	10	1
*Salvelinus leucomaenis*	White-spotted char	1	M94094.1	2	2	1
*Salvelinus malma*	Dolly Varden	7	AB206974.1; AF059901.1-AF059905.1; M94905.1	3	6	1
*Salvelinus malma krasheninnikovi*		1	AF059903.1	3	6	1
*Salvelinus malma lordii*		2	AF059904.1-AF059905.1	3	6	1
*Salvelinus malma malma*		2	AF059901.1-AF059902.1	3	6	1
*Salvelinus namaycush*	Lake trout	2	AF073711.1; M94906.1	3	7	2
*Salmo salar*	Atlantic salmon	2	AF201312.1; HQ260440.1	4	6	5
*Salmo trutta*	Brown trout	4	AF434298.1-AF434301.1	4	6	5
*Oncorhynchus clarkii*	Cutthroat trout	1	AY125136.1	7	7	4
*Oncorhynchus gorbuscha*	Pink salmon	2	AF170533.1; AF308735.1	7	10	4
*Oncorhynchus keta*	Chum salmon	1	AB524075.1	7	8	4
*Oncorhynchus kisutch*	Coho salmon	1	AF097563.1	7	8	5
*Oncorhynchus mykiss*	Rainbow trout	2	AF170533.1; AF308735.1	7	7	4
*Oncorhynchus nerka*	Sockeye salmon	1	AF097561.1	7	8	4
*Oncorhynchus tshawytscha*	Chinook salmon	1	AF170534.1	7	8	5

Candidate primers were obtained *in silico* by screening the genetic sequence data that we generated (accessions MH341972.1 –MH342001.1; S1 Table) and that we obtained from GenBank (Table 1) with *DECIPHER* [16] in R 3.3.2 [17]. The primers were visually aligned with ITSI sequences in MEGA 7.0 [18], and primer lengths and positions were adjusted to optimize annealing temperatures and maximize the number of nucleotide mismatches with non-target sequences. Within the amplicon sequence, a probe binding site unique to bull trout in ITSI was visually identified, and a TaqMan probe with a 6-carboxyfluorescein (FAM)-labeled 5’ end and a minor-groove-binding, non-fluorescent quencher (MGBNFQ; Life Technologies, Grand Island, NY, USA) was designed. The annealing temperatures of the primer and probe sequences were evaluated using Primer Express 3.0.1 (Life Technologies, Grand Island, NY, USA), and potential secondary structure formation was assessed using the IDT OligoAnalyzer (https://www.idtdna.com/calc/analyzer). To confirm the specificity of each primer and probe sequence *in silico*, nucleotide BLAST searches (https://blast.ncbi.nlm.nih.gov/Blast.cgi) of the entire NCBI nucleotide collection database were performed using default parameters. In addition, the potential for non-target amplification was assessed with Primer-BLAST [19] using the forward and reverse primer sequences and the entire NCBI nucleotide collection.

To confirm specificity of the bull trout ITSI assay *in vitro*, qPCR was performed with DNA extracted from tissues of bull trout and 32 non-target species (S2 Table). This DNA was extracted as described above for sequencing. Each DNA extract was analyzed with the bull trout ITSI assay in 15-μl reactions consisting of 7.5 μl of 2X Environmental Master Mix 2.0 (Life Technologies Grand Island, NY, USA), 0.75 μl of 20X bull trout ITSI assay (primers at 900 nM each, and probe at 250 nM), 4 μl of DNA extract (~0.4 ng), and 2.75 μl of PCR-grade distilled water. All experiments were performed on a StepOne Plus Real-time PCR Instrument (Life Technologies, Grand Island, NY, USA) or a QuantStudio 3 Real-time PCR System (Life Technologies, Grand Island, NY, USA) using the manufacturer recommended thermocycling conditions of initial denaturation at 95°C for 10 min and 45 cycles of denaturation at 95°C for 15 s and annealing and extension at 60°C for 1 min. After confirming the specificity of the assay, primer concentrations were optimized following methods outlined in Wilcox et al. [20]. Briefly, the qPCR recipe was prepared with different concentrations (100, 300, 600, and 900 nM) of forward and reverse primers, for a total of 16 different concentration combinations. Each combination was analyzed in triplicate reactions using the cycling conditions described above. The primer concentrations producing the earliest cycle threshold (Ct) value and highest end-point fluorescence were selected for further analysis.

The efficiency and sensitivity of the optimized assay was then assessed by analyzing a seven-level standard curve dilution created from purified qPCR product. Bull trout ITSI was amplified with the assay and purified using the GeneJET PCR Purification Kit (ThermoFisher Scientific, Waltham, MA, USA). The purified product was then quantified on a Qubit 2.0 Fluorometer (ThermoFisher Scientific, Waltham, MA, USA), and serially diluted into sterile TE to create a seven-level standard curve (31 250, 6 250, 1 250, 250, 50, 10, and 2 copies per reaction). Each dilution was analyzed in six replicates using the optimized marker concentrations (forward primer at 600 nM, reverse primer at 900 nM, probe at 250 nM) and qPCR cycling conditions above.

We assessed potential primer competition by comparing the ability of the ITSI assay to detect bull trout DNA in the presence of high concentrations of non-target charr DNA. Extracted DNA of bull trout, Dolly Varden, lake trout, and brook trout was quantified and diluted to 0.1 ng/μl. Bull trout DNA was then diluted into each non-target extract at ratios of 1:1, 1:10, 1:100, and 1:1000. Bull trout DNA was also diluted into sterile TE at the same ratios. Each dilution was analyzed in triplicate reactions.

To test for efficacy when applied *in vivo* to environmental samples, the ITSI assay described here was used to re-analyze eDNA samples that were collected in association with the Range-Wide Bull Trout eDNA Project [6] and were initially analyzed for the presence of bull trout DNA using the *cyt b* assay [4]. For assay validation, four samples each from Idaho, Oregon, and Washington, two of which were positive and two negative for bull trout during the initial analysis, and 14 eDNA samples from Montana, (nine positive and five negative in initial analyses) were selected (S3 Table). The samples were collected following the protocol developed by Carim et al. [21], in which 5 l of water were pumped through a glass microfiber filter (pore size 1.5 μm), and the filter was folded and stored in silica desiccant until further processing. Samples were extracted in a room designated only for this purpose using the DNeasy Blood & Tissue Kit (Qiagen, Inc. Valencia, CA, USA) following a modified protocol described in Carim et al. [22]. The extracts were analyzed in triplicate 15-μl reactions using the same PCR recipe above except using the optimized primer concentrations in the 20X bull trout ITSI assay. During the initial analyses with the Wilcox et al. [4] *cyt b* assay, all eDNA samples were analyzed with a TaqMan Exogenous Internal Positive Control (Life Technologies, Grand Island, NY, USA) and it was determined that none of the samples were inhibited (S3 Table). All qPCR analyses were performed using the cycling conditions described above and were prepared inside a hood where pipettes, tube racks, and consumables were exposed to UV light for at least 1 h. Furthermore, a positive control consisting of tissue-derived bull trout DNA template (starting concentration = 0.1 ng/μl), and a negative control substituting distilled water for DNA template were included with every eDNA analysis.

Finally, the relative detectability of the ITSI and *cyt b* assays when applied to eDNA samples was tested and compared. To do this, 42 eDNA samples associated with the Range-Wide Bull Trout eDNA Project [6] in which bull trout DNA was detected with the *cyt b* assay [4] were re-analyzed with both bull trout assays on the same qPCR plate (S4 Table). The experiment was designed with 14 samples which originally amplified in one of the three replicates, 14 which amplified in two of three replicates, and 14 which amplified in all three replicates. For the ITSI assay, the same qPCR recipe and cycling conditions described for eDNA analysis above were used for this experiment. For the *cyt b* assay, the only difference is that the forward and reverse primers were each in concentrations of 900 nm. All analyses were performed on a StepOne Plus Real-time PCR Instrument. Each eDNA sample was analyzed in three replicates with both the *cyt b* and ITSI assays. The number of wells with positive amplification as well as the Ct values at which fluorescence was observed were compared between the assays. In addition, the Ct values of the tissue-derived positive controls were examined.

For these samples, we also estimated the relative copy number of the *cyt b* and ITSI genes using the resulting Ct values and a standard curve for each assay. We created a standard curve using a linear, double-stranded gBlocks® Gene Fragment (Integrated DNA Technologies, Coralville, IA) that contained the amplicon sequence encompassing each assay region on the same fragment. The gBlock was re-suspended in TE solution, quantified on a Qubit 2.0 Fluorometer (ThermoFisher Scientific, Waltham, MA, USA), and serially diluted to the same concentrations described for assessing the assay sensitivity above. We analyzed the seven-level curve in triplicate reactions with each assay using the same PCR recipe and conditions described above for testing eDNA samples. Using the linear regression from each standard curve, we estimated the relative number of DNA copies of each gene in each eDNA sample (reported as DNA copies per liter of water filtered) and the positive controls (reported as DNA copies per reaction).

## Results

The eDNA assay targeted a 172-nucleotide fragment of the bull trout ITSI (Table 2). The *in silico* nucleotide BLAST indicated that the primers and probe are unique to bull trout, and the Primer-BLAST suggested that bull trout were the only species that would potentially amplify with the primers. The *in vitro* tests confirmed this specificity; the assay detected DNA in all bull trout tissue samples and did not amplify DNA from any non-target tissue samples, including those from other species of chars (S2 Table). Results from the standard curve analysis showed that the assay was efficient (91.2%, *r*^*2*^ = 0.991, slope = -3.55, y-intercept = 38.93) with a limit of detection (defined as the lowest concentration with >95% amplification success; [23]) at 10 ITSI copies per reaction. Nevertheless, the assay can be regarded as even more sensitive, as it detected bull trout DNA in four of six replicates with average concentrations as low as two copies per reaction. There was no evidence of primer competition with the ITSI assay and DNA templates of Dolly Varden, lake trout, and brook trout (S1 Fig). The eDNA samples selected for assay validation of the ITSI assay reproduced the previous results: all samples that were positive for bull trout during initial analysis using the Wilcox et al. [4] *cyt b* eDNA assay were positive for bull trout when analyzed with the ITSI assay, and all samples that were negative for bull trout during the initial analyses were also negative when analyzed with the ITSI assay (S3 Table).

**Table 2:** Primers and probe sequences, estimated annealing temperatures (Tm), and optimal primer concentrations of the eDNA assay for bull trout ITSI. Optimal primer concentrations refer to the lowest concentration of primers resulting in the earliest Ct while maintaining a high end-point fluorescence. The assay amplifies a 172-nucleotide fragment of the bull trout ITSI gene.

Assay component	Sequence (5’-3’)	Tm (°C)	Optimal concentration (nM)
Forward primer	TTCCTTTTGCCTAGGGTAGCG	59.4	600
Reverse primer	CGATACTCAACACGCTTCACAATT	59.2	900
Probe	FAM-CCACGGCCACACGG-MGBNFQ	69	250

**Fig 1:**
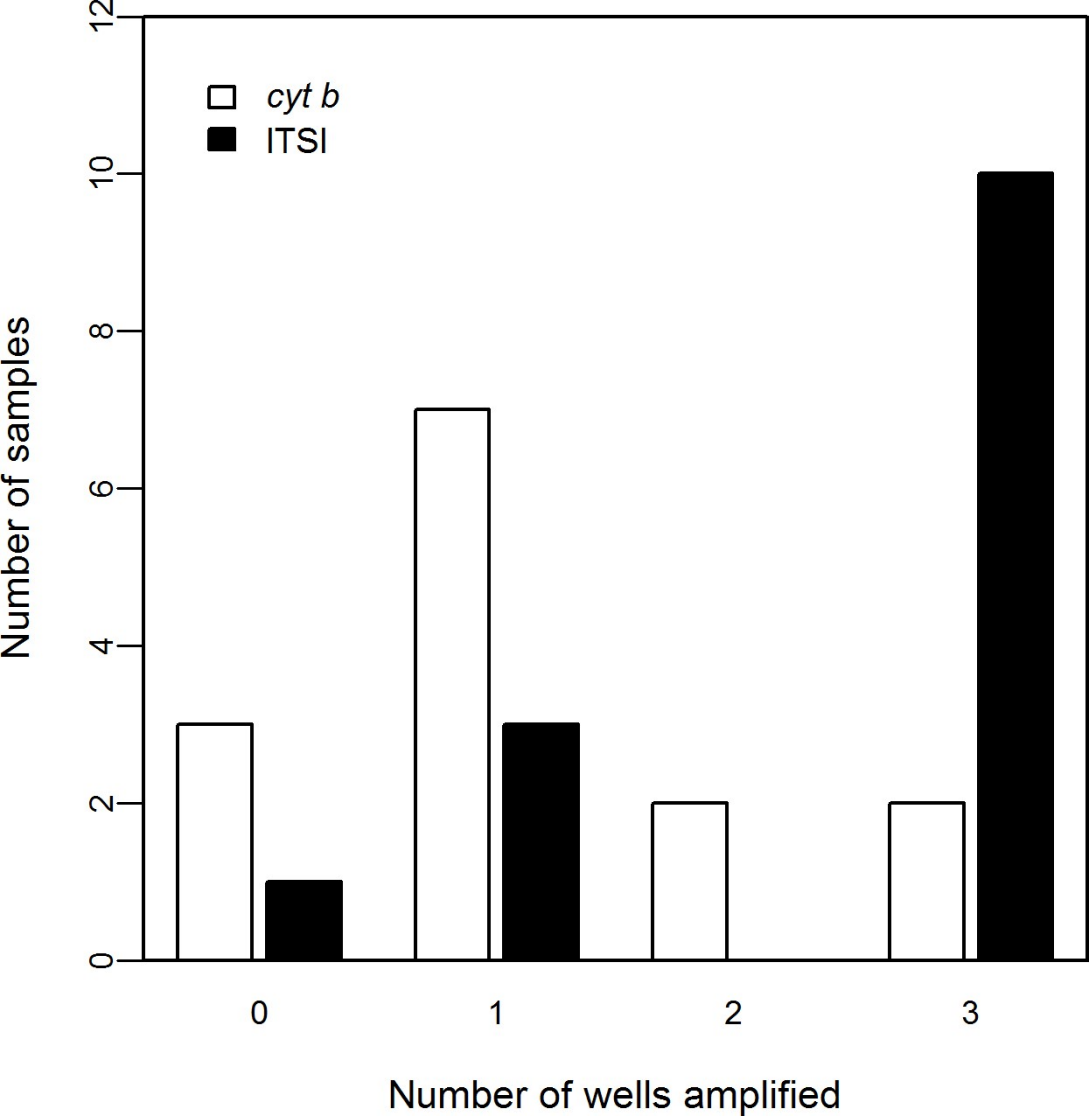
Results of testing environmental DNA samples with assays based in *cyt b* and ITSI. Fourteen samples from the Range-Wide Bull Trout eDNA Project in the northwestern United States [6] that originally amplified in one of three replicates were retested using the same *cyt b* assay and a newly designed assay based in ITSI. The ITSI assay had fewer false negatives and more samples that amplified in all three wells when compared to the *cyt b* based assay.

When comparing bull trout detections of the ITSI and *cyt b* assays, the ITSI assay amplified in more wells and at lower Ct values than did the *cyt b* assay, particularly for samples containing small quantities of eDNA. For the 14 eDNA samples that originally amplified in one of three wells, the follow-up tests resulted in positive detections in 40.5% of the reactions using the *cyt b* assay and 78.6% using the ITSI assay. There were on average nine times as many ITSI DNA copies as *cyt b* in this group of samples (S4 Table). The tissue-derived positive control for this group of samples was detected at a mean Ct of 27.4 (SD = 0.02; 1 320.0 mean copies per reaction, SD = 16.4) with the *cyt b* assay and 23.3 (SD = 0.11; 21 426.9 mean copies per reaction, SD = 1 574.6) with the ITSI assay. For the group of samples that originally amplified in two of three wells, positive detections occurred in 52.4% and 71.4% of the wells with the *cyt b* and ITSI assays, respectively. There were on average 18.6 times as many ITSI DNA copies as *cyt b* in this group of samples. Mean Ct values for the positive control were again lower for ITSI (22.8, SD = 0.12; 28 530.6 mean copies per reaction, SD = 2 273.3) than for *cyt b* (26.9, SD = 0.01; 1 824.4 mean copies per reaction, SD = 16.4). Finally, for the group of samples that originally amplified in all three wells, positive detections occurred in 81.0% and 90.5% of the reactions with the *cyt b* and ITSI assays, respectively. There were on average 12.1 times as many ITSI DNA copies as *cyt b* in this group of samples. The positive control for this group of samples was detected at a mean Ct of 26.4 (SD = 0.06; 2 580.9 mean copies per reaction, SD = 101.3) with the *cyt b* assay, and mean Ct of 22.4 (SD = 0.11; 36 803.8 mean copies per reaction, SD = 2 642.5) with the ITSI assay (S4 Table). For those samples that originally amplified at a single well, retesting with ITSI produced fewer false negatives than *cyt b* (1 vs. 3, respectively), and more samples that amplified at all three wells (Fig 1). The gBlock standard curves for estimating the copy-numbers above resulted in an efficiency = 99.6% (*r*^*2*^ = 0.997, slope = -3.33, y-intercept = 37.79) for the *cyt b* assay and in an efficiency = 93.0% (*r*^*2*^ = 0.989, slope = -3.50, y-intercept = 38.42) for the ITSI assay.

## Discussion

The ITSI assay performed better than the *cyt b* assay from several perspectives. First, it was more specific because it did not detect Dolly Varden DNA or exhibit measurable cross-amplification of lake trout DNA, permitting direct interpretation of the sampling results across more of the range of bull trout without the use of a blocking primer. The ITSI assay also produced higher detection rates in eDNA samples. Assuming Poisson expectations, samples that on average amplify in one of three wells will produce false negative results for 36.8% of samples, i.e., no wells will exhibit positive detections despite the potential presence of low levels of DNA. In our retests of such samples, 21.4% produced false negative results when using the *cyt b* assay, somewhat better than expected. Yet only 7.1% were false negative detections when using the ITSI assay. Moreover, that the majority (71.4%, 10/14, Fig 1) of the samples that originally amplified in a single well using the *cyt b* assay amplified in all three wells when retested using the ITSI assay, indicated that the ITSI assay was much more effective for detecting bull trout. This was further demonstrated by the earlier Ct values associated with the ITSI assay in both eDNA samples and tissue-derived positive controls.

The greater detection rates associated with ITSI were consistent with the findings of Minamoto et al. [12]; one would expect higher copy numbers and therefore higher detection rates in field applications. In addition, the results of our relative comparison of copy-numbers of each gene suggest that ITSI is more abundant than *cyt b* in eDNA samples and tissue-derived DNA positive controls. It is also possible that assays for this region may be more temporally sensitive because of the more rapid degradation of nuclear DNA [13, 24; but see 14]. This might be advantageous for applications in which recognizing rapid changes habitat occupancy is important, such as evaluations of treatments intended to remove nonnative species. Internal transcribed spacers, however, are not a panacea for the development of superior eDNA assays. Although ITS genes are widely used in phylogenies and species identification of plants and fungi [25–26], sequences from animals are much rarer in online databases compared to sequences from the mitochondrial genome such as *cyt b*. Thus, the initial *in silico* screening of candidate assays must be preceded by much more extensive sampling and sequencing of sympatric or closely related taxa than needed for the more commonly used mitochondrial genes. Moreover, not all copies of the internal transcribed spacers within an individual are identical [27–28]. Variation in amplification among ribotypes may lead to more uncertainty in detection efficiency or when relating eDNA quantity to animal abundance. It is also important to note that ribosomal copy numbers are a function of genome size [29], so interspecific detection efficiencies are likely to vary, and direct comparison of species-to-species eDNA quantities would be ill-advised. This variation is already evident among the three species that have been tested (common carp *Cyprinus carpio*, [12]; Macquarie perch *Maquaria australasica*, [30]; bull trout, this study). Nonetheless, development of nuclear ribosomal assays offers a useful alternative to practitioners and researchers that use eDNA sampling to address questions about species presence.

## Supporting information

S1 Table Sample information for ITSI sequence data generated for developing the improved bull trout eDNA assay including species, sample size (n), country or state (UNK if unknown), waterbody, sequence length (number of nucleotides), and GenBank accession number. Total samples: Arctic char (*n* = 1), brook trout (*n* = 10), bull trout (*n* = 8), Dolly Varden (*n* = 8), and lake trout (*n* = 3).
(DOCX)

S2 Table Species, sample size (n), and detection results (y = detected; n = not detected) of *in vitro* testing of the bull trout ITSI eDNA assay. Origin refers to state or province from which the specimens were collected, or is designated as ‘f’ for farmed origin.
(DOCX)

S3 Table Collection information and detection results (y = yes, detected; n = not detected) for eDNA samples used for *in vivo* testing of the bull trout ITS1 eDNA assay. Also included are the mean Ct values of the Internal Positive Control (mean Ct IPC) for each eDNA sample and the respective negative control (mean Ct IPC Neg) from the initial analysis with the *cyt b* assay. Samples were considered inhibited (n = not inhibited) if there was a shift in the mean Ct of the IPC of any given eDNA sample when compared to the Ct value of the IPC in the PCR negative control.
(DOCX)

S4 Table Collection information for eDNA samples used for comparing relative detection efficiencies of samples that amplified in one, two, or three replicate reactions during a prior analysis with the *cyt b* (Wilcox et al. 2013) assay. Environmental DNA samples were re-analyzed with the *cyt b* assay and the ITS1 assay described here and the mean DNA copies/liter (SD = standard deviation) for each respective amplicon were estimated.
(DOCX)

S1 Fig Assessment of primer competition for the ITSI assay when bull trout DNA is in the presence of high concentrations of DNA from closely related charr. Part A shows the amplification curves of the ITSI assay when bull trout is diluted into sterile TE at 1:1, 1:10, 1:100, and 1:1000. Part B shows the amplification curves of the ITSI assay when bull trout is diluted into Dolly Varden DNA solution at 1:1, 1:10, 1:100, and 1:1000. Part C shows the amplification curves of the ITSI assay when bull trout is diluted into lake trout DNA solution at 1:1, 1:10, 1:100, and 1:1000. Part D shows the amplification curves of the ITSI assay when bull trout is diluted into brook trout DNA solution at 1:1, 1:10, 1:100, and 1:1000.
(PDF)
