# Supplementary material for: An improved environmental DNA assay for bull trout (Salvelinus confluentus) based on the ribosomal internal transcribed spacer I
Source: PLoS One. 2018 Nov 6;13(11):e0206851. doi: 10.1371/journal.pone.0206851 (PMC6219789; doi:10.1371/journal.pone.0206851)
Supplement: S1 Table — Total samples: Arctic char (n = 1), brook trout (n = 10), bull trout (n = 8), Dolly Varden (n = 8), and lake trout (n = 3). (DOCX) [file pone.0206851.s001.docx]

**S1 Table. Sample information for ITSI sequence data generated for developing the improved bull trout eDNA assay including species, sample size (n), country or state (UNK if unknown), waterbody, sequence length (number of nucleotides), and GenBank accession number. Total samples: Arctic char (*n* = 1), brook trout (*n* = 10), bull trout (*n* = 8), Dolly Varden (*n* = 8), and lake trout (*n* = 3).**

| **Species name** | **Common name** | **n** | **Origin** | **Waterbody** | **Sequence length** | **GenBank accession** |
| --- | --- | --- | --- | --- | --- | --- |
| Salvelinus alpinus | Arctic char | 1 | Norway | Unknown | 576 | MH341972 |
| Salvelinus confluentus | Bull trout | 1 | ID | Dewey Creek | 576 | MH341973 |
|  |  | 1 | MT | Morrell Creek | 580 | MH341977 |
|  |  | 1 | MT | WF Trout Creek | 580 | MH341980 |
|  |  | 1 | OR | Sun Creek | 578 | MH341978 |
|  |  | 1 | OR | Upper MF Willamette River | 580 | MH341979 |
|  |  | 2 | WA | Diablo Reservoir | 580 | MH341974–MH341975 |
|  |  | 1 | WA | Gorge Reservoir | 580 | MH341976 |
| Salvelinus fontinalis | Brook trout | 1 | ID | EF Weiser River | 567 | MH341986 |
|  |  | 1 | MT | Avalanche Gulch | 567 | MH341981 |
|  |  | 2 | MT | Bostwick Creek | 567 | MH341983– MH341984 |
|  |  | 1 | MT | EF Moose River | 567 | MH341985 |
|  |  | 2 | MT | Little Blackfoot River | 567 | MH341987– MH341988 |
|  |  | 1 | MT | Warm Springs Creek | 567 | MH341990 |
|  |  | 1 | VA | Little River | 567 | MH341989 |
|  |  | 1 | WA | Beaver pond near SF Snoqualmie River | 567 | MH341982 |
| Salvelinus malma | Dolly Varden trout | 1 | AK | Gulf of Alaska | 568 | MH341992 |
|  |  | 1 | AK | Lower Taku River | 578 | MH341993 |
|  |  | 1 | WA | Diablo Reservoir | 578 | MH341991 |
|  |  | 1 | WA | Dungeness River | 342 | MH341997 |
|  |  | 1 | WA | Mink Creek | 575 | MH341994 |
|  |  | 1 | WA | Skagit River | 578 | MH341995 |
|  |  | 2 | WA | Sol Duc River | 341; 574 | MH341996; MH341998 |
| Salvelinus namaycush | Lake trout | 1 | OR | Lake Pend Oreille | 571 | MH342000 |
|  |  | 1 | WA | Lake Chelan | 571 | MH341999 |
|  |  | 1 | UNK | Lake Superior | 561 | MH342001 |
