# Supplementary material for: An improved environmental DNA assay for bull trout (Salvelinus confluentus) based on the ribosomal internal transcribed spacer I
Source: PLoS One. 2018 Nov 6;13(11):e0206851. doi: 10.1371/journal.pone.0206851 (PMC6219789; doi:10.1371/journal.pone.0206851)
Supplement: S2 Table — Origin refers to state or province from which the specimens were collected, or is designated as ‘f’ for farmed origin. (DOCX) [file pone.0206851.s002.docx]

**S2 Table. Species, sample size (n), and detection results (y = detected; n = not detected) of *in vitro* testing of the bull trout ITSI eDNA assay. Origin refers to state or province from which the specimens were collected, or is designated as ‘f’ for farmed origin.**

|  | **Family name** | **Species name** | **Common name** | **n** | **Detected (y/n)** | **Origin** |
| --- | --- | --- | --- | --- | --- | --- |
| Targets | Salmonidae | *Salvelinus confluentus* | Bull trout | 1 | y | Ant Basin Creek, ID |
|  |  |  |  | 1 | y | Dewey Creek, ID |
|  |  |  |  | 1 | y | Sheep Creek, ID |
|  |  |  |  | 1 | y | Wesley Creek, ID |
|  |  |  |  | 1 | y | East Fork Rock Creek, MT |
|  |  |  |  | 1 | y | Grave Creek, MT |
|  |  |  |  | 1 | y | Long North Fork Creek, MT |
|  |  |  |  | 1 | y | Morrell Creek, MT |
|  |  |  |  | 1 | y | Red Ives Creek, MT |
|  |  |  |  | 1 | y | Squaw Creek, MT |
|  |  |  |  | 1 | y | Swan Lake, MT |
|  |  |  |  | 1 | y | West Fork Trout Creek, MT |
|  |  |  |  | 1 | y | Wounded Buck Creek, MT |
|  |  |  |  | 2 | y | Middle Fork Willamette River, OR |
|  |  |  |  | 1 | y | Sun Creek, OR |
|  |  |  |  | 2 | y | Diablo Reservoir, WA |
|  |  |  |  | 2 | y | Gorge Reservoir, WA |
| Non-targets | Centrarchidae | *Micropterus dolomieu* | Smallmouth bass | 1 | n | North Fork John Day River, OR |
|  |  | *Micropterus salmoides* | Largemouth bass | 1 | n | Missouri River, MT |
|  | Cottidae | *Cottus cognatus* | Slimy sculpin | 1 | n | Yukon territory |
|  | Cyprinidae | *Cyprinus carpio* | Common carp | 1 | n | Glendo Reservoir, WY |
|  |  | *Gila atraria* | Utah chub | 1 | n | Henry’s Lake, ID |
|  |  | *Rhinichthys cataractae* | Longnose dace | 1 | n | Tin Cup Creek, ID |
|  | Esocidae | *Esox lucius* | Northern pike | 1 | n | Big Pine Creek, AK |
|  |  | *Esox maquinongy* | Muskellunge | 1 | n | Leech Lake, MN |
|  | Gasterosteidae | *Gasterosteus aculeatus* | Three spine stickleback | 1 | n | Unnamed tributary of Snoqualmie River, WA |
|  | Ictaluridae | *Ictalurus punctatus* | Channel catfish | 1 | n | Missouri River, MT |
|  | Lotidae | *Lota lota* | Burbot | 1 | n | Missouri River, MT |
|  | Percidae | *Perca flavescens* | Yellow perch | 1 | n | Lake Washington, WA |
|  |  | *Sander vitreus* | Walleye | 1 | n | Lake Washington, WA |
|  | Petromyzontidae | *Entosphenus tridentatus* | Pacific lamprey | 1 | n | Imnaha River, OR |
|  |  | *Lampetra richardsoni* | Western brook lamprey | 1 | n | Hills Creek Reservoir, OR |
|  | Salmonidae | *Coregonus clupeaformis* | Lake whitefish | 1 | n | Sherburne Reservoir, MT |
|  |  | *Oncorhynchus clarkii clarkii* | Coastal cutthroat trout | 1 | n | Fifteen Mile Creek, OR |
|  |  | *Oncorhynchus gorbuscha* | Pink salmon | 1 | n | Bonneville Fish Hatchery, OR |
|  |  | *Oncorhynchus keta* | Chum salmon | 1 | n | Columbia River, OR |
|  |  | *Oncorhynchus kisutch* | Coho salmon | 1 | n | Macaulay Salmon Hatchery, AK |
|  |  |  |  | 1 | n | Klamath River, CA |
|  |  |  |  | 1 | n | Quilcene National Fish Hatchery, WA |
|  |  | *Oncorhynchus mykiss* | Rainbow trout | 1 | n | Ennis National Fish Hatchery, MT |
|  |  |  |  | 1 | n | Annie Creek, OR |
|  |  |  |  | 1 | n | Cherry Creek, WA |
|  |  | *Oncorhynchus nerka* | Sockeye salmon | 1 | n | Oxbow Fish Hatchery, OR |
|  |  | *Oncorhynchus tshawytscha* | Chinook salmon | 1 | n | Panther Creek, ID |
|  |  |  |  | 1 | n | Fifteen Mile Creek, OR |
|  |  | *Prosopium williamsoni* | Mountain whitefish | 1 | n | Rock Creek, MT |
|  |  | *Salmo salar* | Atlantic salmon | 1 | n | f |
|  |  | *Salmo trutta* | Brown trout | 1 | n | Annie Creek, OR |
|  |  | *Salvelinus alpinus* | Arctic char | 1 | n | Norway |
|  |  | *Salvelinus fontinalis* | Brook trout | 1 | n | East Fork Weiser River, ID |
|  |  |  |  | 2 | n | Bostwick Creek, MT |
|  |  |  |  | 2 | n | East Fork Moose Creek, MT |
|  |  |  |  | 2 | n | Little Blackfoot River, MT |
|  |  |  |  | 1 | n | South Fork Snoqualmie River, WA |
|  |  | *Salvelinus malma* | Dolly Varden trout | 4 | n | Taku River, AK |
|  |  |  |  | 3 | n | Diablo Reservoir, WA |
|  |  |  |  | 3 | n | Dungeness River, WA |
|  |  |  |  | 3 | n | Gorge Reservoir, WA |
|  |  |  |  | 3 | n | Mink Creek, WA |
|  |  |  |  | 3 | n | Skagit River, WA |
|  |  |  |  | 3 | n | Sol Duc River, WA |
|  |  | *Salvelinus namaycush* | Lake trout | 1 | n | Cosley Lake, MT |
|  |  |  |  | 1 | n | Saint Mary Lake, MT |
|  |  |  |  | 2 | n | Lake Chelan, WA |
|  |  | *Thymallus arcticus* | Arctic grayling | 1 | n | LeMarche Creek, MT |
|  |  | *Thymallus thymallus* | Grayling | 1 | n | Norway |
