# Supplementary material for: An improved environmental DNA assay for bull trout (Salvelinus confluentus) based on the ribosomal internal transcribed spacer I
Source: PLoS One. 2018 Nov 6;13(11):e0206851. doi: 10.1371/journal.pone.0206851 (PMC6219789; doi:10.1371/journal.pone.0206851)
Supplement: S3 Table — Also included are the mean Ct values of the Internal Positive Control (mean Ct IPC) for each eDNA sample and the respective negative control (mean Ct IPC Neg) from the initial analysis with the cyt b assay. Samples were considered inhibited (n = not inhibited) if there was a shift in the mean Ct of the IPC of any given eDNA sample when compared to the Ct value of the IPC in the PCR negative control. (DOCX) [file pone.0206851.s003.docx]

**S3 Table. Collection information and detection results (y = yes, detected; n = not detected) for eDNA samples used for *in vivo* testing of the bull trout ITS1 eDNA assay. Also included are the mean Ct values of the Internal Positive Control (mean Ct IPC) for each eDNA sample and the respective negative control (mean Ct IPC Neg) from the initial analysis with the *cyt b* assay. Samples were considered inhibited (n = not inhibited) if there was a shift in the mean Ct of the IPC of any given eDNA sample when compared to the Ct value of the IPC in the PCR negative control.**

|  |  |  |  |  |  |  |  | **DNA detected** | |
| --- | --- | --- | --- | --- | --- | --- | --- | --- | --- |
| **Waterbody** | **Zone** | **Easting** | **Northing** | **Collection date** | **Mean Ct IPC** | **Mean Ct IPC Neg** | **Inhibited** | ***cytb* assay** | **ITS1 assay** |
| Dollar Creek, ID | 11 | 602691 | 4952327 | 9/27/2016 | 26 | 26.2 | n | n | n |
| Edna Creek, ID | 11 | 609824 | 4869483 | 8/14/2015 | 27.8 | 27.8 | n | **y** | **y** |
| Little Beaver Creek, ID | 11 | 613810 | 4871532 | 10/6/2016 | 28.6 | 28.9 | n | n | n |
| Simmons Creek, ID | 11 | 621341 | 5221665 | 7/7/2015 | 27.9 | 27.9 | n | **y** | **y** |
| Blodgett Creek, MT | 11 | 711381 | 5127712 | 10/19/2016 | 27.5 | 27.6 | n | **y** | **y** |
| Boulder Creek, MT | 11 | 711931 | 5080068 | 11/30/2016 | 27.8 | 27.8 | n | **y** | **y** |
| Clifford Creek, MT | 12 | 294759 | 5089028 | 10/10/2016 | 27.7 | 27.9 | n | **y** | **y** |
| Coal Creek, MT | 11 | 707420 | 5058785 | 9/10/2016 | 26.6 | 28.7 | n | n | n |
| Divide Creek, MT | 12 | 279462 | 5104894 | 8/30/2016 | 28.6 | 28.7 | n | **y** | **y** |
| East Fork Camp Creek, MT | 12 | 272422 | 5067732 | 9/2/2016 | 28.7 | 28.8 | n | n | n |
| Fred Burr Creek, MT | 11 | 710440 | 5137178 | 9/15/2016 | 27.8 | 27.8 | n | n | n |
| Moose Creek, MT | 12 | 289869 | 5095383 | 8/31/2016 | 28.8 | 28.8 | n | **y** | **y** |
| Sheephead Creek, MT | 11 | 693814 | 5069570 | 9/10/2016 | 27.7 | 27.9 | n | **y** | **y** |
| Skalkaho Creek, MT | 12 | 275333 | 5116919 | 8/17/2016 | 27.3 | 27.5 | n | **y** | **y** |
| Soda Springs Creek, MT | 11 | 701982 | 5078404 | 9/11/2016 | 27.8 | 27.8 | n | n | n |
| Watchtower Creek, MT | 11 | 696353 | 5075269 | 9/11/2016 | 27.8 | 27.8 | n | **y** | **y** |
| Weasel Creek, MT | 12 | 279520 | 5112339 | 8/30/2016 | 28.6 | 28.7 | n | **y** | **y** |
| West Fork Butte Creek, MT | 11 | 699386 | 5177830 | 10/17/2016 | 27.4 | 27.6 | n | n | n |
| Chapman Creek, OR | 11 | 634881 | 4888358 | 9/9/2015 | 28.0 | 27.9 | n | **y** | **y** |
| Trapper Creek, OR | 10 | 576980 | 4826127 | 9/14/2015 | 27.8 | 27.6 | n | **y** | **y** |
| Unnamed Tributary to SF Gold Creek, OR | 11 | 586806 | 4945545 | 8/31/2015 | 27.7 | 27.7 | n | n | n |
| Unnamed Tributary to Third Fork Squaw Creek, OR | 11 | 563566 | 4918370 | 8/19/2015 | 27.1 | 27.2 | n | n | n |
| Clear Creek, WA | 10 | 580849 | 5118107 | 10/12/2016 | 27.8 | 27.9 | n | n | n |
| Indian Creek, WA | 10 | 646764 | 5316123 | 10/10/2016 | 28.1 | 28.3 | n | n | n |
| Leland Creek, WA | 10 | 646175 | 5280224 | 9/13/2016 | 26 | 26.1 | n | **y** | **y** |
| Unnamed Tributary to Pine Creek, WA | 10 | 572403 | 5106075 | 10/26/2016 | 27.7 | 27.6 | n | **y** | **y** |
