# Supplementary material for: An improved environmental DNA assay for bull trout (Salvelinus confluentus) based on the ribosomal internal transcribed spacer I
Source: PLoS One. 2018 Nov 6;13(11):e0206851. doi: 10.1371/journal.pone.0206851 (PMC6219789; doi:10.1371/journal.pone.0206851)
Supplement: S4 Table — Environmental DNA samples were re-analyzed with the cyt b assay and the ITS1 assay described here and the mean DNA copies/liter (SD = standard deviation) for each respective amplicon were estimated. (DOCX) [file pone.0206851.s004.docx]

**S4 Table. Collection information for eDNA samples used for comparing relative detection efficiencies of samples that amplified in one, two, or three replicate reactions during a prior analysis with the *cyt b* (Wilcox et al. 2013) assay.** Environmental DNA samples were re-analyzed with the *cyt b* assay and the ITS1 assay described here and the mean DNA copies/liter (SD = standard deviation) for each respective amplicon were estimated.

|  |  |  |  |  |  | **Mean DNA copies/liter (SD)** | |
| --- | --- | --- | --- | --- | --- | --- | --- |
|  | **Waterbody** | **Zone** | **Easting** | **Northing** | **Collection date** | ***cyt b*** | **ITSI** |
| eDNA samples that initially amplified in one replicate with the *cyt b* assay | Clear Creek | 11 | 490284 | 4980153 | 10/7/2016 | 0 | 22.6 (8.4) |
|  | Dave Creek | 11 | 635722 | 4642756 | 9/16/2016 | 2.3 (4.1) | 41.1 (24.1) |
|  | French Creek | 10 | 648232 | 5274577 | 7/19/2016 | 6.2 (6.3) | 47.5 (15.0) |
|  | Gold Creek | 11 | 622171 | 5224613 | 7/7/2015 | 2.1 (3.6) | 19.0 (13.7) |
|  | Jack Creek | 10 | 656120 | 5273027 | 8/1/2016 | 1.1 (1.9) | 21 (7.8) |
|  | Jarbridge River | 11 | 632034 | 4630498 | 9/17/2016 | 21.7 (8.3) | 94.5 (9.4) |
|  | Lake Fork Creek | 11 | 504504 | 4985166 | 10/6/2016 | 0 | 0 |
|  | Little Blackfoot River | 12 | 385526 | 5141851 | 8/19/2015 | 1.6 (2.8) | 3.1 (5.4) |
|  | Quartz Creek | 11 | 613385 | 5230943 | 7/9/2015 | 1.2 (2.1) | 4.6 (8.1) |
|  | SF Rock Creek | 12 | 294321 | 5108948 | 8/20/2016 | 1.0 (1.7) | 2.7 (4.6) |
|  | Sherlock Creek | 11 | 636980 | 5213647 | 9/11/2015 | 1.1 (1.9) | 46.6 (13.6) |
|  | Simmons Creek | 11 | 622176 | 5222108 | 7/7/2015 | 5.4 (6.9) | 87.2 (10.2) |
|  | Simmons Creek | 11 | 626018 | 5222513 | 9/29/2015 | 0 | 37.7 (10.8) |
|  | South Boulder Creek | 12 | 330955 | 5141559 | 9/29/2016 | 7.5 (6.6) | 34.1 (36.5) |
| eDNA samples that initially amplified in two replicates with the *cyt b* assay | Boulder Creek | 12 | 337869 | 5136359 | 10/4/2016 | 0 | 4.0 (7.0) |
|  | Dave Creek | 11 | 636247 | 4641908 | 9/16/2016 | 5.7 (4.9) | 264.6 (31.8) |
|  | Duck Creek | 11 | 504501 | 4990045 | 10/7/2016 | 1.7 (1.6) | 0 |
|  | East Pine Creek | 11 | 493290 | 4981357 | 10/20/2016 | 0 | 893.2 (30.9) |
|  | French Creek | 10 | 650146 | 5276904 | 7/18/2016 | 8.3 (4.0) | 67.7 (19.6) |
|  | Gold Creek | 11 | 621406 | 5223870 | 7/7/2015 | 2.8 (4.8) | 46.8 (5.6) |
|  | Jack Creek | 10 | 656896 | 5274599 | 7/28/2016 | 17.2 (7.6) | 181.0 (70.7) |
|  | Little Blackfoot River | 12 | 386581 | 5138867 | 8/19/2015 | 8.6 (8.5) | 1.5 (2.5) |
|  | Little Fish L Creek | 12 | 293929 | 5107719 | 8/20/2016 | 2.5 (4.4) | 2.9 (1.0) |
|  | Quartz Creek | 11 | 613302 | 5230568 | 7/9/2015 | 1.5 (2.5) | 1.1 (2.0) |
|  | Sherlock Creek | 11 | 636094 | 5213374 | 9/11/2015 | 2.9 (5.1) | 25.4 (8.0) |
|  | Simmons Creek | 11 | 624444 | 5222855 | 7/7/2015 | 19.7 (17.1) | 23.4 (16.6) |
|  | Simmons Creek | 11 | 628606 | 5221783 | 9/24/2015 | 1.4 (2.5) | 0 |
|  | West Fork Pine Creek | 11 | 630218 | 4631571 | 9/18/2016 | 13.5 (0.9) | 85.4 (9.3) |
| eDNA samples that initially amplified in three replicates with the *cyt b* assay | Clear Creek | 11 | 490028 | 4978306 | 10/7/2016 | 0 | 2.7 (4.6) |
|  | Dave Creek | 11 | 636485 | 4640991 | 9/16/2016 | 35.4 (3.4) | 1105.7 (69.7) |
|  | East Pine Creek | 11 | 492398 | 4981574 | 10/20/2016 | 6.9 (4.7) | 1323.4 (106.1) |
|  | French Creek | 10 | 649377 | 5276277 | 7/21/2016 | 2.6 (2.7) | 116.8 (21.9) |
|  | Gold Creek | 11 | 620816 | 5223176 | 7/7/2015 | 3.6 (3.1) | 16.1 (2.4) |
|  | Jack Creek | 10 | 656390 | 5273957 | 8/1/2016 | 10.1 (7.1) | 24.2 (7.6) |
|  | Little Blackfoot River | 12 | 385891 | 5139475 | 8/19/2015 | 6.9 (7.8) | 1.4 (2.4) |
|  | Quartz Creek | 11 | 612928 | 5229412 | 7/9/2015 | 2.2 (2.1) | 4.5 (2.8) |
|  | Ross Fork | 12 | 288224 | 5106552 | 8/16/2016 | 126.8 (28.2) | 473.9 (49.6) |
|  | Sherlock Creek | 11 | 635294 | 5213711 | 9/11/2015 | 31.1 (6.4) | 94.6 (22.9) |
|  | Simmons Creek | 11 | 623510 | 5222906 | 7/7/2015 | 9.7 (11.3) | 14.9 (3.8) |
|  | Simmons Creek | 11 | 626676 | 5221810 | 9/29/2015 | 4.3 (5.0) | 18.3 (11.4) |
|  | South Boulder Creek | 12 | 330067 | 5139666 | 9/29/2016 | 17.0 (8.5) | 133.1 (27.1) |
|  | West Fork Pine Creek | 11 | 628895 | 4627979 | 9/18/2016 | 71.4 (22.9) | 632.5 (25.8) |
