# Supplementary figures and images for: An improved environmental DNA assay for bull trout (Salvelinus confluentus) based on the ribosomal internal transcribed spacer I
Source: PLoS One. 2018 Nov 6;13(11):e0206851. doi: 10.1371/journal.pone.0206851 (PMC6219789; doi:10.1371/journal.pone.0206851)

**A**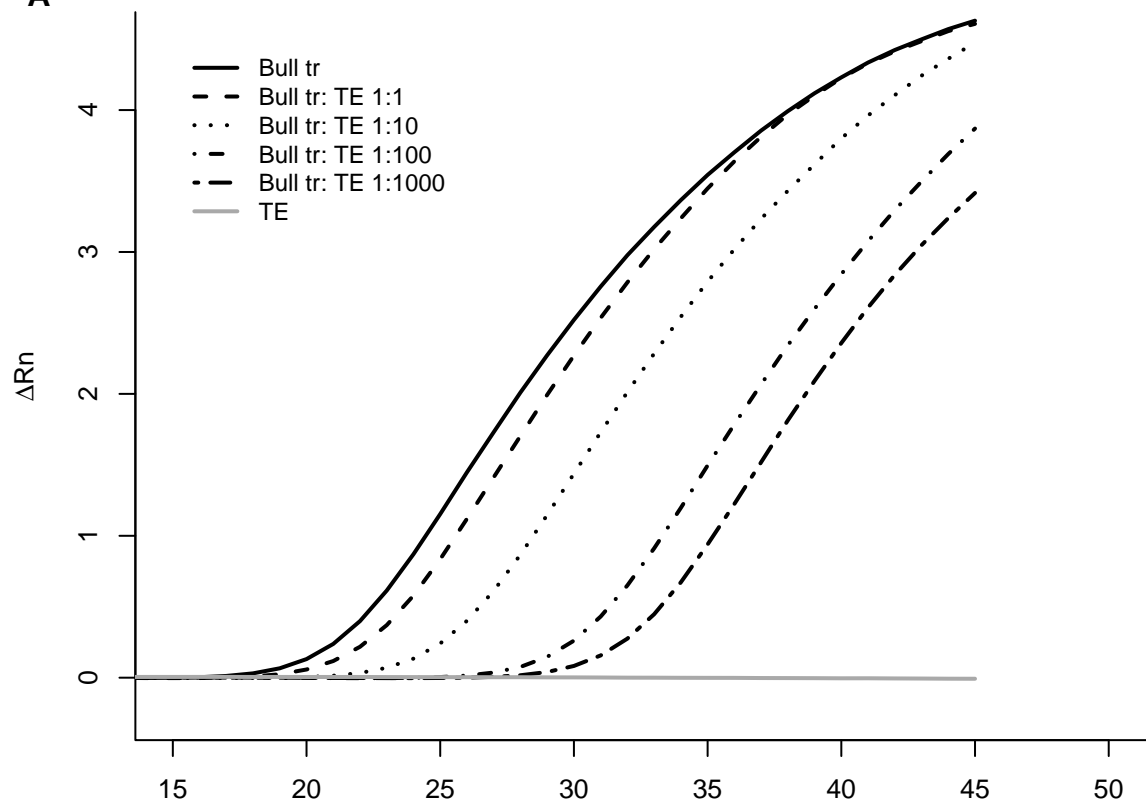**B**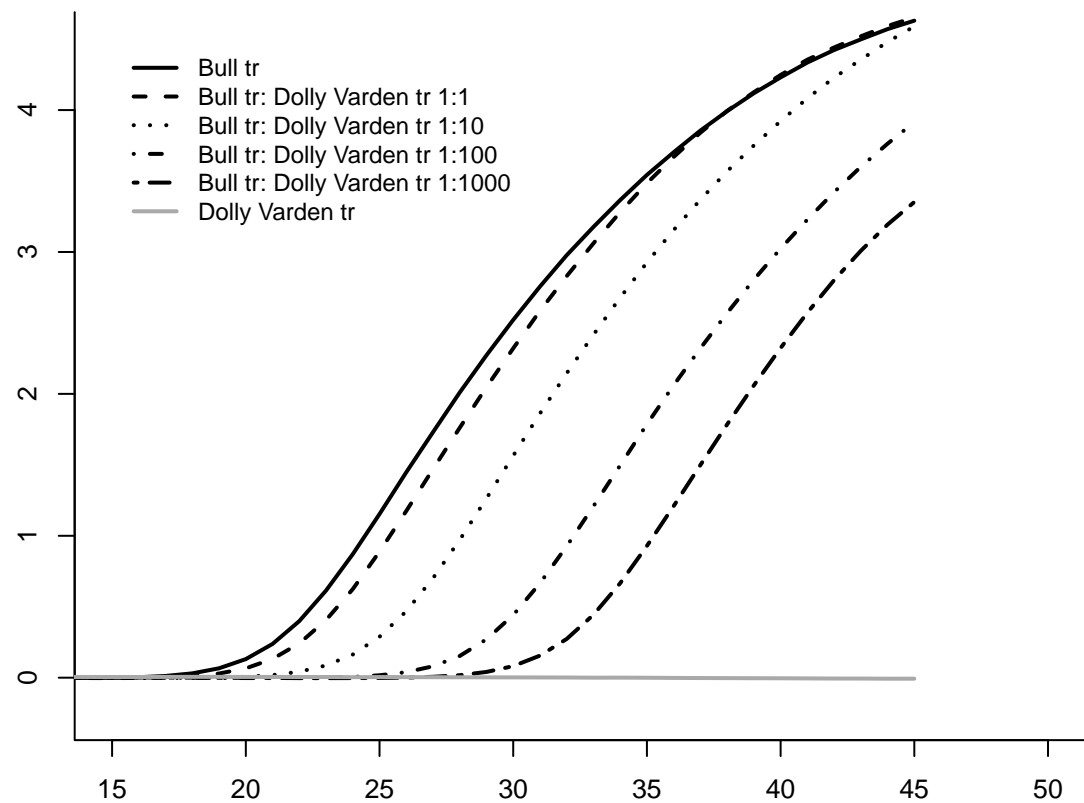**C**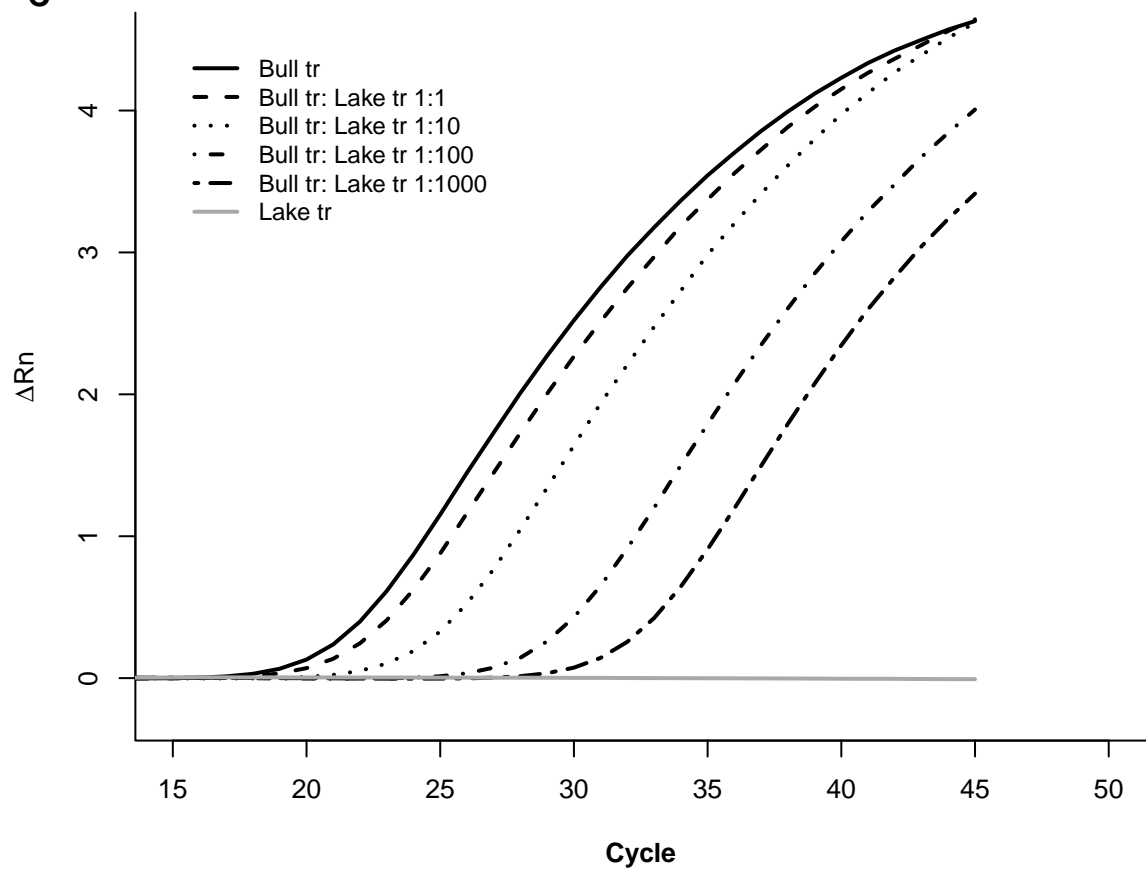**D**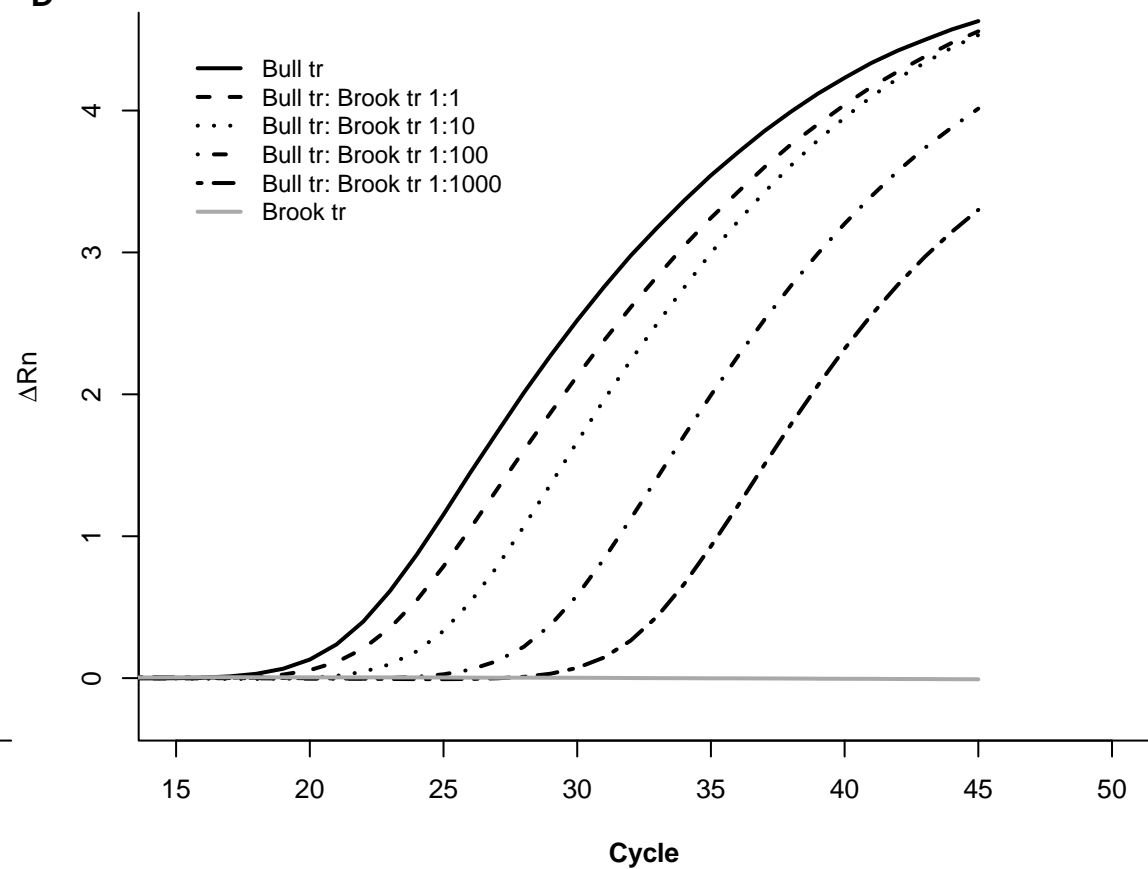

Supplement: S1 Fig — Part A shows the amplification curves of the ITSI assay when bull trout is diluted into sterile TE at 1:1, 1:10, 1:100, and 1:1000. Part B shows the amplification curves of the ITSI assay when bull trout is diluted into Dolly Varden DNA solution at 1:1, 1:10, 1:100, and 1:1000. Part C shows the amplification curves of the ITSI assay when bull trout is diluted into lake trout DNA solution at 1:1, 1:10, 1:100, and 1:1000. Part D shows the amplification curves of the ITSI assay when bull trout is diluted into brook trout DNA solution at 1:1, 1:10, 1:100, and 1:1000. (PDF) [file pone.0206851.s005.pdf]
